# Innovative approaches in physical education: leveraging cognitive activation to boost student outcomes

**DOI:** 10.3389/fpsyg.2025.1481381

**Published:** 2025-07-07

**Authors:** Haoran Zha, Xia Ding, Wenye Li

**Affiliations:** ^1^School of Physical Education and Health Management, Chongqing University of Education, Chongqing, China; ^2^Institute of Education, Nanjing University, Nanjing, China

**Keywords:** cognitive activation teaching strategies, physical education, classroom engagement, self-efficacy, health behaviors, primary school students

## Abstract

**Introduction:**

Physical education (PE) often struggles with suboptimal student engagement, which impedes the development of physical competence and lifelong health habits. This challenge is especially acute in under-resourced areas like Southwest China. Cognitive Activation Teaching Strategies (CATS), which promote higher-order thinking, offer a potential solution. This study, therefore, aimed to investigate the impact of CATS on primary school students’ physical performance and health behaviors, specifically examining the mediating roles of classroom engagement, self-efficacy, motivation, and emotional regulation.

**Methods:**

A mixed-methods sequential explanatory design was employed. First, quantitative data were collected from a stratified sample of 929 primary school students and their parents in Southwest China using validated questionnaires. The hypothesized mediation model was then analyzed using Structural Equation Modeling (SEM). Following this, qualitative data were gathered through semi-structured interviews and non-participant classroom observations with 12 purposively selected “high-CATS” teachers to provide deeper insight into the classroom mechanisms at play.

**Results:**

The SEM results indicated that CATS significantly and positively predicted students’ physical performance and health behaviors. This relationship was strongly mediated by classroom engagement, PE motivation, and particularly, PE self-efficacy. Conversely, the pathway from CATS to emotional regulation was not statistically significant, and emotional regulation did not significantly predict health behaviors. The qualitative findings corroborated the quantitative data, revealing that teachers’ use of goal-setting, progressive challenges, and feedback created a “productive struggle,” which visibly enhanced students’ intrinsic motivation and collaborative engagement.

**Discussion:**

This study provides robust evidence that cognitive activation is a highly effective pedagogical approach in PE. By fostering self-efficacy and motivation, CATS directly enhance in-class engagement and physical performance. The findings suggest that designing PE tasks to be cognitively challenging is crucial for improving student outcomes. However, the limited impact on emotional regulation and out-of-class health behaviors indicates that CATS alone may be insufficient. Educational programs should therefore integrate CATS to boost classroom success while also developing comprehensive, multi-faceted interventions to cultivate emotional skills and promote the transfer of healthy habits beyond the school setting.

## 1 Introduction

Physical education (PE) in primary schools is crucial for the holistic development of children, fostering not only physical health but also social, emotional, and cognitive growth. Regular physical activity during childhood is associated with a myriad of benefits, including improved cardiovascular health, enhanced mood and mental well-being, and superior academic performance (Kliziene et al., 2021; Faigenbaum et al., 2015; Wang et al., 2024). Despite these well-documented advantages, current teaching methods often fail to fully engage students or motivate sustained participation (Manninen and Campbell, 2022), leading to suboptimal outcomes in both physical fitness and educational attainment (Harvey et al., 2020).

In Southwest China, these challenges are further exacerbated by socio-economic dis-parities (Zhang et al., 2023; Shen et al., 2024). Uneven economic development in this region has led to significant differences in the availability and quality of educational resources, including those for physical education. Students in Southwest China have limited access to high-quality PE resources and facilities compared to their peers in more affluent coastal regions (Yang et al., 2025). This disparity not only hampers their physical development but also poses broader challenges for their overall well-being and academic success. Moreover, teachers in this region often face difficulties in implementing effective teaching strategies, resulting in less favorable classroom behaviors and outcomes among students. Confronting these constraints, the Chinese Government has repositioned school PE as a strategic lever for public-health equity in policy roadmaps such as the Outline for Building a Leading Sports Nation and the National Fitness Plan (2021–2025), while successive Five-Year education programs explicitly prioritize resource transfers to the western region (Liu and Dehao, 2019). Echoing this national agenda, Sichuan, Yunnan, Guizhou and Chongqing have issued dedicated action plans that emphasize facility renewal, teacher deployment and stronger community-school linkages. Accordingly, the success of these resource-focused initiatives ultimately hinges on complementary pedagogical innovations that can translate enhanced inputs into meaningful gains in students’ engagement, learning, and health outcomes.

Cognitive activation teaching strategies, which emphasize engaging and thought-provoking activities, offer a promising solution to these challenges by promoting active learning and critical thinking. Although this approach has been validated in various educational settings, including large-scale assessments like PISA, it has yet to be fully integrated into physical education. This study aims to address this gap by investigating the impact of cognitive activation teaching strategies on primary school students’ behaviors and performance in PE classes.

The primary objectives of this study are to: (1) evaluate the impact of these teaching strategies on students’ classroom engagement, physical performance, and health behaviors. (2) Explore the possible mechanisms through which these teaching strategies influence student outcomes. (3) Explore in depth how this mechanism occurs at the level of classroom learning.

By focusing on these objectives, this study seeks to provide valuable insights into the effectiveness of cognitive activation teaching strategies in the context of physical education, particularly within the unique socio-economic landscape of Southwest China.

## 2 Literature review and research hypotheses

### 2.1 Cognitive activation teaching strategies

Cognitive activation teaching strategies are instructional methods designed to engage students in higher-order thinking processes, promoting deep understanding and active learning. These strategies emphasize stimulating students’ cognitive functions through challenging and thought-provoking tasks, encouraging them to apply, analyze, and evaluate information in various contexts, including physical education. Key characteristics of cognitive activation include the use of open-ended questions, problem-solving activities, and opportunities for students to articulate their reasoning and reflect on their learning processes. By focusing on these elements, cognitive activation aims to foster critical thinking, creativity, and a deeper grasp of the subject matter, whether it be theoretical concepts or practical skills in physical activities (Li et al., 2021; Heikonen et al., 2017; Weinstein et al., 1981).

Empirical research has consistently demonstrated the positive impact of cognitive activation strategies on student learning outcomes. Data from the Program for International Student Assessment (PISA) highlight that students exposed to cognitively activating instruction tend to perform better academically. PISA results indicate that students who frequently encounter tasks requiring deep cognitive engagement achieve higher scores in reading, mathematics, and science compared to their peers experiencing more traditional, rote-based instruction (Caro et al., 2016). Additionally, studies have shown that cognitive activation enhances student engagement and motivation, making them more interested and invested in their learning. This increased engagement is crucial for fostering a positive learning environment and encouraging lifelong learning habits (McKeachie et al., 1985; Wenzel, 2017).

Although much of the evidence supporting cognitive activation comes from general education settings, its principles can be effectively applied to physical education. By incorporating cognitively engaging activities into PE, educators can promote not only physical skills but also a deeper understanding of health and wellness (Montague, 1997). Cognitive strategy instruction has been shown to improve mathematical problem-solving among middle school students with learning disabilities (Montague and Dietz, 2009). The effectiveness of these strategies has been further supported by research on their application in teaching complex scientific concepts (Tobin et al., 1988). While cognitive activation teaching strategies have demonstrated significant benefits across various academic disciplines, their application in physical education remains largely unexplored. This study seeks to bridge this gap by investigating the impact of cognitive activation teaching strategies on student behaviors and performance in primary school PE classes. Further research is necessary to evaluate their effectiveness in this unique context.

### 2.2 Physical performance and health behaviors (student outcome)

Positive teaching strategies are crucial for enhancing physical performance and promoting healthy behaviors in students. Effective instructional methods in physical education (PE) improve motor skills, physical fitness, and instill long-term health habits. Research indicates that engaging teaching approaches significantly enhance endurance, strength, coordination, and overall physical competence while also fostering regular exercise and balanced nutrition (Ennis, 2017). Additionally, applying Positive Behavior Support (PBS) strategies in PE has been shown to improve student behavior and achievement (Buchanan et al., 2013).

Cognitive activation teaching strategies, which emphasize engaging and thought-provoking activities, have significant potential to promote physical performance and health behaviors. These strategies enhance motivation by making PE classes more interesting, improve emotional regulation by helping students manage physical challenges, and boost self-efficacy by instilling confidence in their abilities (Deci and Ryan, 2000; Gross, 2013; Trudeau and Shephard, 2008). In the Chinese educational context, physical performance includes measurable outcomes such as endurance and coordination, while health behaviors involve regular physical activity and proper nutrition, aligning with national curriculum standards (Department of Basic Education, Ministry of Education, 2022).

Cognitive activation teaching strategies can enhance physical performance and health behaviors in primary school PE classes by focusing on the roles of motivation, emotional regulation, and self-efficacy. The integration of multi-teaching styles and active reflection (MTA) has been shown to significantly improve fitness levels, motor competence, enjoyment, and the amount of physical activity among students (Invernizzi et al., 2019). Furthermore, a systematic review indicates that quality-based PE interventions are associated with improvements in health-related physical fitness and fundamental motor skills (FMS) (García-Hermoso et al., 2020). These findings underscore the importance of adopting diverse and engaging teaching methods to achieve positive learning outcomes in physical education. Based on this, hypothesis 1 and hypothesis 2 are proposed:

*H1*: Cognitive activation teaching has significant positive effects on health behavior.

*H2*: Cognitive activation teaching has significant positive effects on physical performance.

### 2.3 Classroom engagement in PE

Classroom engagement is a crucial mediator between teaching strategies and educational outcomes, particularly in physical education (PE) (Guo et al., 2023). Engaged students exhibit enthusiasm, focus, cooperation, and resilience, correlating with better physical performance and increased motivation (Pitzer and Skinner, 2017; Wu et al., 2013). Research indicates that student engagement in PE is significantly influenced by teaching strategies. For instance, skill practice is positively associated with student engagement, whereas inactive instruction has a negative impact (Bevans et al., 2010).

This study investigates how cognitive activation teaching strategies can enhance engagement in PE, with the goal of improving students’ physical and health outcomes through more dynamic and effective teaching practices. Evidence shows that teaching behaviors such as need-supportive and relatedness support positively influence student engagement in PE (González-Peño et al., 2021). Moreover, cooperative learning models addressing physical, cognitive, and social learning outcomes have been shown to enhance student engagement (Casey and Goodyear, 2015). Structured and supportive teaching methods evidently foster a more engaging and effective PE environment, which in turn promotes better learning and physical activity outcomes.

Thus, positive classroom engagement in PE is essential for translating effective teaching strategies into improved educational outcomes. Research consistently highlights that student engagement mediates the relationship between instructional quality and learning achievements. For example, physically active classrooms are associated with small but significant improvements in academic performance compared to traditional, sedentary classrooms (Bedard et al., 2019). Additionally, the quality of teacher engagement and feedback during active lessons significantly impacts student participation and physical activity levels (Errisuriz et al., 2021). Therefore, implementing cognitive activation strategies and fostering an engaging classroom environment are critical for enhancing learning behaviors in primary school PE, ultimately leading to better physical and educational outcomes.

*H3*: Cognitive activation teaching has a significant positive effect on classroom participation in PE.

*H4-a*: Classroom participation in PE has a significant positive effect on health behavior.

*H4-b*: Classroom participation in PE has a significant positive effect on physical performance.

### 2.4 Potential emotional mechanisms

Emotional mechanisms are critical in linking teaching strategies with student outcomes, particularly in physical education (PE). Positive emotions such as enjoyment and interest are strongly associated with higher levels of classroom engagement. When students experience these emotions, they are more likely to participate actively, pay attention, and persist in tasks (Løvoll et al., 2020). Cognitive activation teaching strategies, designed to make learning more engaging and relevant, can elicit these positive emotions, thereby enhancing student engagement and fostering a supportive learning environment (Simonton and Garn, 2020).

Research across various disciplines, including PE, has consistently shown that emotions significantly impact learning outcomes. Positive emotional states facilitate cognitive processes such as memory, problem-solving, and critical thinking, which are essential for academic and physical performance. Studies indicate that students who enjoy physical activities and feel emotionally supported by their teachers perform better and adopt healthier behaviors (Fredrickson, 2000). In the Chinese educational context, understanding these emotional mechanisms is crucial for developing effective PE programs (Simonton et al., 2017). This study aims to explore how cognitive activation strategies influence student emotions in PE and how these emotions, in turn, affect engagement and learning outcomes, providing insights for creating more effective and enjoyable PE classes (Rahimi and Askari Bigdeli, 2014).

**Figure 1:**
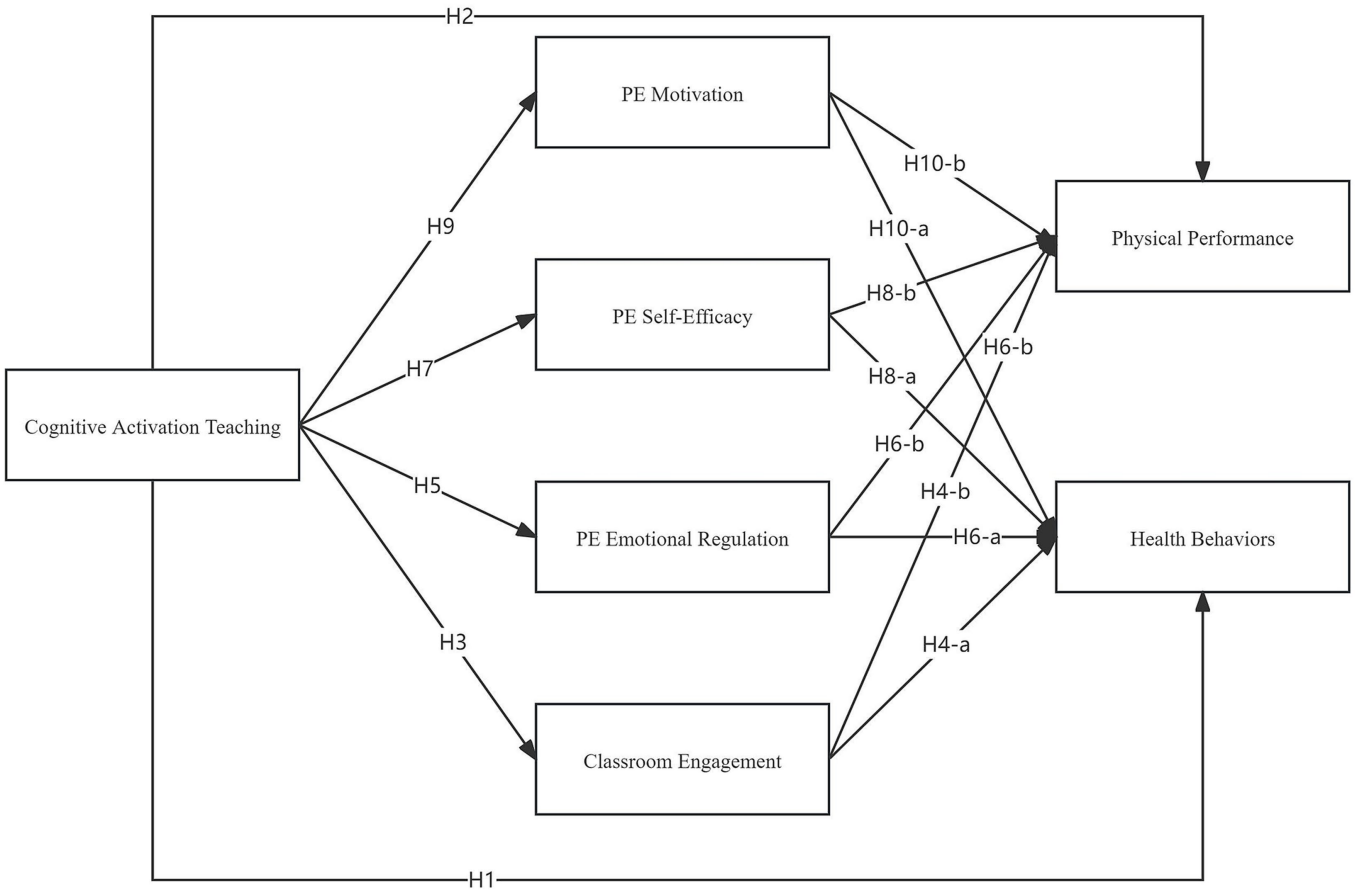
Mediation hypothesis model.

In conclusion, emotional pathways play a significant role in students’ learning processes. Current research emphasizes the importance of positive emotions in enhancing student engagement and learning outcomes but has paid relatively little attention to emotional factors in sports learning through positive teaching methods (Kim and Hamann, 2007) (Figure 1).

*H5*: Cognitive activation teaching has a significant positive effect on PE Emotional Regulation.

*H6-a*: PE Emotional Regulation has a significant positive effect on health behavior.

*H6-b*: PE Emotional Regulation has a significant positive effect on physical performance.

*H7*: Cognitive activation teaching has a significant positive effect on PE Self-Efficacy.

*H8-a*: PE Self-Efficacy has a significant positive effect on health behavior.

*H8-b*: PE Self-Efficacy has a significant positive effect on physical performance.

*H9*: Cognitive activation teaching has a significant positive effect on PE Motivation.

*H10-a*: PE Motivation has a significant positive effect on health behavior.

*H10-b*: PE Motivation has a significant positive effect on physical performance.

## 3 Methodology

### 3.1 Sample and data collection

To capture the pronounced economic and educational heterogeneity that characterizes our study municipality in western China—where highly urbanized districts abut resource-constrained rural townships—we adopted the three-stage stratified unequal-probability design prescribed in the National Compulsory Education Quality Monitoring Scheme (2021, revised) issued by the Ministry of Education of the People’s Republic of China (2021). In Stage 1, all public primary schools were stratified jointly by urban–rural location and enrolment size, two dimensions known to covary with PE infrastructure and instructional capacity. Stage 2 selected schools within each stratum by probability-proportional-to-size (PPS). Stage 3 used simple random sampling to draw one intact Grade-4 class per selected school (modal age ≈ 10 years), thereby paralleling the national benchmark of surveying 30 Grade-4 pupils per site and ensuring every child a known, non-zero inclusion probability (Cochran, 1977). It should be noted here that the questionnaire was divided into two parts, one for students and the other for parents to fill out. This was mainly because some specific sports performance results are difficult to obtain through self-assessment by the students. Therefore, we developed an observation questionnaire for parents to use for evaluation.

Of the 1,000 questionnaires administered, 958 were returned (response rate = 95.8%), and 929 met completeness and procedural-fidelity criteria. The realized sample comprises 52.1% boys and 47.9% girls; 62.9% reside in urban districts and 37.1% in rural townships—figures that mirror the latest local school-age population statistics. In addition, 38.7% are only children, consistent with official demographic records. These distributions confirm that the final dataset is broadly representative of Grade-4 pupils in the municipality. Descriptive details appear in Table 1.

**Table 1:** Demographic information of the participants (sample size = 929).

Demographic variable	Category	Number	Percentage
Gender	Male	484	52.1%
	Female	445	47.9%
Location	Urban	584	62.9%
	Rural	345	37.1%
Only child status	Only child	360	38.7%
	Non-only child	569	61.3%

### 3.2 Research instrument

All constructs were assessed with instruments that build on previously validated Chinese-language measures. Physical Performance and Health Behaviors were operationalized with task sheets derived from the national Physical Education and Health Curriculum Standards, a framework whose scoring rubrics have been validated in multiple domestic studies of primary-school cohorts. Classroom Engagement, Physical Education Motivation, Self-Efficacy and Emotional Regulation were measured with Chinese versions of internationally recognized scales that earlier work has shown to possess satisfactory reliability and construct validity in comparable age groups. Each instrument was subjected to a translation-back-translation check (where required), minor wording adjustments for PE context, and a small-scale pilot to confirm clarity for 10-year-old respondents before full administration.

#### 3.2.1 Cognitive activation teaching strategies

The cognitive activation teaching strategies questionnaire was designed with reference to the OECD’s Programme for International Student Assessment (PISA) framework (OECD, 2019), tailored specifically for physical education (PE). This instrument aims to measure the extent to which PE teaching strategies stimulate higher-order thinking and active engagement among students. The questionnaire includes items that assess the use of open-ended questions, problem-solving tasks, and opportunities for students to reflect on their physical and health-related learning processes. For example, students were asked to rate statements such as, “During PE classes, how often are you encouraged to think about how different exercises affect your body?” and “How often do you solve problems related to physical fitness during PE lessons?” This survey was administered to students, who provided their perceptions of the cognitive activation strategies employed in their PE classes.

#### 3.2.2 Physical performance and health behaviors

The measurement of physical performance and health behaviors was based on the curriculum standards issued by the Chinese Ministry of Education (Department of Basic Education, Ministry of Education, 2022), with modifications informed by prior research (Ennis, 2017; Buchanan et al., 2013). This instrument evaluates students’ physical fitness, motor skills, and health behaviors such as exercise frequency, dietary habits, and overall health awareness, specifically within the context of PE. The questionnaire included items like, “How often does your child participate in physical activities outside of school?” and “How well does your child understand the importance of a balanced diet for their physical health?” Parents were asked to complete this questionnaire, providing insights into their children’s physical performance and health behaviors outside of school. The parent questionnaire will be matched with the student questionnaire based on the student ID in the later data merge.

#### 3.2.3 Classroom engagement

Classroom engagement in PE was measured using a validated scale adapted from prior research (Gong, 2021), tailored to reflect active learning behaviors in physical activities and health education. This instrument assesses students’ active participation and focus during PE classes. Items in the questionnaire were designed to capture how frequently students are engaged in PE activities. Example items include, “How focused are you during PE activities?” and “How often do you actively participate in PE classes?” The questionnaire was administered to students to gauge their level of engagement in PE activities.

#### 3.2.4 Physical education motivation, self-efficacy, and emotional regulation

The questionnaires for measuring physical education motivation, self-efficacy, and emotional regulation were adapted from established instruments in the literature (Lin, 2019; Hao, 2021; Dishman et al., 2010), specifically for the context of PE. Items originally written in English underwent a forward–back translation into Chinese, while items that already existed in Chinese kept their native wording; the resulting bilingual instrument was then scrutinized for content equivalence and clarity by three PhD-level experts in educational assessment, who approved the final version used in this study. These scales assess students’ intrinsic and extrinsic motivation toward physical activity, their confidence in their ability to perform specific physical tasks, and their ability to manage emotions during physical activities. The questionnaires include items such as, “How confident are you in your ability to complete a new physical exercise?” and “How do you manage your emotions when you find a physical activity challenging?” These instruments were administered to students to obtain a comprehensive understanding of these psychological constructs within the context of PE.

### 3.3 Data analysis

The data analysis will begin with Confirmatory Factor Analysis (CFA) to validate the measurement models, ensuring the reliability and accuracy of the constructs. This will be followed by descriptive statistics to summarize the sample characteristics and distributions of the study variables. Finally, Structural Equation Modeling (SEM) will be employed to test the hypothesized relationships and mediation effects among cognitive activation teaching strategies, physical performance, health behaviors, and psychological constructs.

To complement and further elucidate the quantitative findings, we will conduct follow-up interviews with teachers and students. These interviews aim to provide deeper insights into the observed relationships and mediation effects, particularly focusing on how cognitive activation teaching strategies influence classroom engagement, self-efficacy, motivation, and emotional regulation, and how these factors subsequently impact physical performance and health behaviors. This qualitative approach will enrich our understanding by capturing the experiences and perspectives of those directly involved in the educational process.

## 4 Results

### 4.1 Descriptive statistics

Table 2 reports the mean values, standard deviation values, maximum and minimum values. The descriptive statistics for the seven core variables indicate generally positive responses from both students and parents. Cognitive Activation Teaching Strategies (CATS), Classroom Engagement (CE), PE Motivation (PEM), PE Self-Efficacy (PES), and PE Emotional Regulation (PEEM) were assessed through student questionnaires. CATS had a mean score of 2.95 (SD = 1.02) on a scale of 1 to 4, suggesting moderate use of these strategies in PE classes. CE had a mean score of 3.94 (SD = 1.02) on a scale of 1 to 5, reflecting strong student engagement in PE activities. PEM, PES, and PEEM, measured on a scale from 1 to 5, had mean scores of 4.28 (SD = 0.81), 3.96 (SD = 0.90), and 4.15 (SD = 0.81), respectively, indicating high levels of motivation, confidence in physical tasks, and effective emotional regulation among students.

**Table 2:** Descriptive statistics.

Construct	*N*	Mean	Std. deviation	Minus	Max
Cognitive activation teaching strategies (CATS)	929	2.95	1.02	1.00	4.00
Physical performance (PP)	929	3.19	0.86	1.00	4.00
Health behaviors (HB)	929	4.04	1.06	1.00	5.00
Classroom engagement (CE)	929	3.94	1.02	1.00	5.00
PE motivation (PEM)	929	4.28	0.81	1.00	5.00
PE self-efficacy (PES)	929	3.96	0.9	1.00	5.00
PE emotional regulation (PEEM)	929	4.15	0.81	1.00	5.00

Physical Performance (PP) and Health Behaviors (HB) were evaluated through parent questionnaires. PP had a mean score of 3.19 (SD = 0.86) on a scale of 1 to 4, indicating that parents generally rated their children’s physical performance positively. HB, with a mean score of 4.04 (SD = 1.06) on a scale of 1 to 5, suggests that parents observed their children engaging in healthy behaviors frequently.

Overall, the data suggest that students frequently engage in cognitively activating PE strategies and demonstrate positive physical and psychological outcomes, while parents also report favorable physical performance and health behaviors among their children.

### 4.2 Confirmatory factor analysis

The results of the Confirmatory Factor Analysis (CFA) for the seven core variables indicate satisfactory validity and reliability across all constructs (shown in Table 3). The internal consistency, measured by Cronbach’s Alpha, ranged from 0.759 to 0.877, all exceeding the critical standard of 0.7. The Kaiser–Meyer–Olkin (KMO) measure of sampling adequacy for all variables was above 0.8, confirming the suitability of the data for factor analysis.

**Table 3:** Confirmatory factor analysis and model fitting.

Construct	Alpha	KMO	GFI	RMSEA	RMR	CFI	NFI	NNFI
Critical standard	>0.7	>0.8	>0.9	<0.1	<0.05	>0.9	>0.9	>0.9
Cognitive activation teaching strategies (CATS)	0.759	0.853	0.925	0.083	0.041	0.952	0.931	0.941
Physical performance (PP)	0.78	0.876	0.937	0.074	0.033	0.962	0.943	0.955
Health behaviors (HB)	0.821	0.907	0.958	0.069	0.028	0.977	0.964	0.963
Classroom engagement (CE)	0.766	0.824	0.909	0.092	0.038	0.948	0.929	0.931
PE motivation (PEM)	0.807	0.881	0.91	0.075	0.039	0.953	0.944	0.942
PE self-efficacy (PES)	0.877	0.948	0.962	0.089	0.023	0.906	0.961	0.976
PE emotional regulation (PEEM)	0.812	0.892	0.944	0.073	0.031	0.958	0.947	0.952

Fit indices further supported the adequacy of the measurement models. The Goodness-of-Fit Index (GFI) values ranged from 0.909 to 0.962, all above the critical threshold of 0.9, indicating good model fit. The Root Mean Square Error of Approximation (RMSEA) values were all below 0.1, with the highest at 0.092 for Classroom Engagement (CE), still within acceptable limits. The Root Mean Square Residual (RMR) values were all below 0.05, supporting the adequacy of the models.

Comparative Fit Index (CFI) values ranged from 0.906 to 0.977, Normed Fit Index (NFI) values from 0.929 to 0.964, and Non-Normed Fit Index (NNFI) values from 0.931 to 0.976, all surpassing the critical standard of 0.9. These indices collectively demonstrate that the measurement models for Cognitive Activation Teaching Strategies (CATS), Physical Performance (PP), Health Behaviors (HB), Classroom Engagement (CE), PE Motivation (PEM), PE Self-Efficacy (PES), and PE Emotional Regulation (PEEM) are reliable and valid for use in further analyses.

### 4.3 Hypothesis testing

The path hypothesis testing results elucidate the relationships between cognitive activation teaching strategies (CATS), classroom engagement (CE), physical education emotional regulation (PEEM), self-efficacy (PESE), motivation (PEM), health behaviors (HB), and physical performance (PP). The hypotheses and their outcomes are systematically presented below and shown in Table 4.

**Table 4:** Hypotheses testing result.

Hypothesis	Hypothesized path	Estimate	Sig	Hypothesis testing result
H1	CAT→HB	0.087	***	Supported
H2	CAT→PP	0.133	***	Supported
H3	CAT→CE	0.270	***	Supported
H4-a	CE → HB	0.139	***	Supported
H4-b	CE → PP	0.142	***	Supported
H5	CAT→PEER	0.039	0.183	Unsupported
H6-a	PEER→HB	0.122	0.097	Unsupported
H6-b	PEER→PP	0.175	***	Supported
H7	CAT→PESE	0.216	***	Supported
H8-a	PESE→HB	0.209	***	Supported
H8-b	PESE→PE	0.310	***	Supported
H9	CAT→PEM	0.287	***	Supported
H10-a	PEM → HB	0.178	0.239	Unsupported
H10-b	PEM → PP	0.128	***	Supported

***indicates significance, where *represents *p* < 0.05, **represents *p* < 0.01, and ***represents *p* < 0.001.

The analysis indicates that cognitive activation teaching strategies (CATS) positively influence health behaviors (HB) and physical performance (PP), with significant path estimates of 0.087 (*p* < 0.001) and 0.133 (*p* < 0.001), respectively, thus supporting H1 and H2. Additionally, these strategies were found to significantly enhance classroom engagement (CE), as evidenced by the path estimate of 0.270 (*p* < 0.001), supporting H3.

Further, classroom engagement (CE) has a positive impact on both health behaviors (HB) and physical performance (PP), with significant path estimates of 0.139 (*p* < 0.001) and 0.142 (*p* < 0.001), respectively, thus supporting H4-a and H4-b. These findings underscore the importance of student engagement in PE classes for promoting better health and performance outcomes.

However, the path from cognitive activation teaching strategies (CATS) to emotional regulation in PE (PEEM) was not significant, with an estimate of 0.039 (*p* = 0.183), indicating that these strategies do not significantly affect emotional regulation in PE, and thus, H5 is not supported. Similarly, the path from emotional regulation in PE (PEEM) to health behaviors (HB) was also not significant, with an estimate of 0.122 (*p* = 0.097), showing that emotional regulation does not significantly influence health behaviors, and thus, H6-a is not supported. In contrast, the path from emotional regulation in PE (PEEM) to physical performance (PP) was significant, with an estimate of 0.175 (*p* < 0.001), indicating that better emotional regulation in PE positively impacts physical performance, supporting H6-b.

The results further reveal that cognitive activation teaching strategies (CATS) positively influence self-efficacy in PE (PESE), with a significant path estimate of 0.216 (*p* < 0.001), thus supporting H7. Self-efficacy in PE (PESE) significantly impacts both health behaviors (HB) and physical performance (PP), with path estimates of 0.209 (*p* < 0.001) and 0.310 (*p* < 0.001), respectively, thus supporting hypotheses H8-a and H8-b. These findings highlight the critical role of self-efficacy in achieving positive health and performance outcomes in PE.

Lastly, cognitive activation teaching strategies (CATS) were found to significantly enhance motivation in PE (PEM), with a path estimate of 0.287 (*p* < 0.001), thus supporting H9. However, while the path from motivation in PE (PEM) to health behaviors (HB) was not significant, with an estimate of 0.178 (*p* = 0.239), indicating no significant effect on health behaviors and thus not supporting H10-a, the path from motivation in PE (PEM) to physical performance (PP) was significant, with an estimate of 0.128 (*p* < 0.001), thus supporting H10-b. This suggests that higher motivation in PE is crucial for improved physical performance.

**Figure 2:**
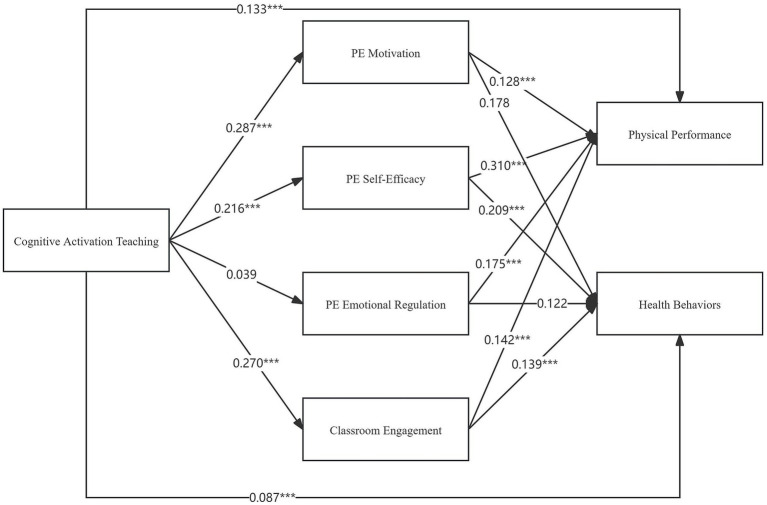
Final model result. ***indicates significance, where *represents *p* < 0.05, **represents *p* < 0.01, and ***represents *p* < 0.001.

Overall, the findings demonstrate that cognitive activation teaching strategies (CATS) significantly enhance classroom engagement (CE), self-efficacy (PESE), and motivation (PEM), which in turn positively affect physical performance (PP) and, to a lesser extent, health behaviors (HB). However, the influence of emotional regulation (PEEM) on these outcomes is more nuanced, indicating areas for further exploration. The final model results are shown in Figure 2.

### 4.4 Qualitative interviews

To probe the classroom mechanisms through which cognitive activation teaching strategies (CATS) operate, we implemented a criterion-based purposive sampling procedure nested within the quantitative frame described above (Patton, 2014). First, all PE teachers (*N* = 74) in the 24 schools that formed our survey sample were invited to complete a short screening questionnaire containing a 12-item CATS-frequency scale (*α* = 0.88). Teachers whose composite scores lay in the top quintile were flagged as “high-CATS” candidates. In the second stage, we sought maximum variation in school context by selecting one centrally located urban school (enrolment ≈ 1,800; seven PE staff), one peri-urban school (≈1,100; five PE staff) and one rural township school (≈750; four PE staff). Each of the three sites had at least four high-CATS teachers who also expressed willingness to host observations, so all were retained, giving a final qualitative sample of 12 teachers—four per school. All interviewees provided written informed consent for both semi-structured interviews (40–55 min) and non-participant classroom observations (two lessons each), which spanned regular PE periods, indoor health modules and one live-streamed online lesson per teacher. Data collection continued until thematic saturation was achieved (Guest et al., 2006). Geographically, the three schools represent the municipality’s urban core, an industrial satellite town and an outlying mountainous county, thereby capturing the full spectrum of economic and educational conditions without disclosing identifiable locations. All the teachers who participated in the interviews and observations had at least a bachelor’s degree in physical education or related fields. The average teaching experience was 8.3 years, and all were teachers who had been employed for more than 3 years. There were no obvious teaching adaptation problems.

#### 4.4.1 Teacher strategies for enhancing motivation, engagement, and self-efficacy

We conducted 12 face-to-face semi-structured interviews (40–55 min each) with the observed high-CATS teachers in quiet rooms on school premises immediately after two lesson observations per teacher; all sessions were audio-recorded, transcribed verbatim within 48 h and supplemented by standardized observation field-notes. Using NVivo 14, transcripts and notes were subjected to a hybrid deductive-inductive reflexive thematic analysis: *a priori* codes anchored in core CATS constructs guided initial coding, while open coding allowed unanticipated strategies to surface (Braun and Clarke, 2006; Braun and Clarke, 2021); iterative theme development, constant comparison and integration of field-notes ensured analytic coherence.

The interviews revealed that teachers used a variety of strategies aimed at boosting motivation, engagement, and self-efficacy in a PE context. To enhance motivation, teachers crafted fitness challenges and skill-building exercises that were both demanding and attainable, allowing students to experience success and build a sense of accomplishment. Goal-setting activities were commonly integrated, where students set personal fitness targets, such as improving their mile run time or mastering a new skill, and tracked their progress. Engagement was fostered through interactive and problem-solving tasks, such as team sports and group exercises, requiring active participation and collaboration. For self-efficacy, teachers provided regular constructive feedback during physical activities and created opportunities for students to succeed in progressively more difficult tasks, thereby building their confidence and competence in physical abilities. Table 5 shows the common teaching behaviors reported by teachers when using cognitive activation in teaching and their specific directions.

**Table 5:** An overview of teachers’ teaching behavior in cognitive activation teaching.

Teaching behavior/method	Description	Teaching focus
Goal-setting activities	Students set personal fitness targets (e.g., improving mile run time) and track their progress using charts or fitness apps.	Motivation, self-efficacy
Fitness challenges	Structured challenges like a weekly push-up or sit-up competition to improve fitness.	Motivation, engagement
Skill-building exercises	Progressive exercises such as dribbling drills in soccer, with increasing difficulty.	Self-efficacy, engagement
Team sports and group exercises	Activities like basketball or volleyball that require teamwork and collaboration.	Engagement, motivation
Problem-solving tasks	Interactive tasks such as obstacle courses that require strategy and critical thinking.	Engagement, self-efficacy
Constructive feedback	Regular, specific feedback during activities (e.g., correcting posture during a squat) to improve techniques and build confidence.	Self-efficacy
Relay races	Competitive and collaborative running activities like baton relay races to enhance engagement.	Engagement
Technique demonstrations	Teachers model proper techniques, such as showing the correct way to shoot a basketball.	Self-efficacy
Peer encouragement	Encouraging students to support and motivate each other during activities, like cheering during a race.	Motivation, engagement
Use of technology	Incorporating fitness apps or online tools to track progress and set goals, such as using pedometers or heart rate monitors.	Motivation, engagement

#### 4.4.2 Students’ active learning behavior in PE

Classroom observations and teacher reports highlighted significant changes in student behavior following the implementation of CATS. During goal-setting activities, students were observed discussing their personal fitness targets and strategies to achieve them, demonstrating a clear investment in their physical progress. Interactive tasks, such as relay races and team sports, involved students working together with high levels of participation and enthusiasm. Teachers frequently offered constructive feedback during activities, such as demonstrating proper techniques in a soccer drill or encouraging students during a challenging workout, visibly boosting students’ confidence and willingness to engage with new challenges.

Teachers reported that these strategies led to noticeable improvements in students’ active learning behaviors. Students demonstrated increased intrinsic motivation, as evidenced by their active engagement in setting and striving toward personal fitness goals, such as improving their sprint times or achieving a certain number of push-ups. Their participation in PE classes rose markedly, with more students actively collaborating and engaging with their peers during physical activities. Self-efficacy improved as students successfully completed challenging physical tasks and received positive feedback, reinforcing their confidence in their physical abilities. Overall, the implementation of CATS in PE classes created a more dynamic, motivated, and engaged learning environment, aligning with the quantitative findings of this study. Specific examples and detailed teacher feedback on these changes will be elaborated on in the discussion section.

## 5 Discussion

The findings from this study offer significant insights into the complex relationships between cognitive activation teaching strategies, classroom engagement, self-efficacy, motivation, emotional regulation, health behaviors, and physical performance in the context of physical education. Through a rigorous analysis combining quantitative data with qualitative interviews, this research not only validates established theories but also uncovers new dimensions in the pedagogical approaches to physical education. The discussion below delves into these relationships, providing a comprehensive interpretation of the results, their alignment with existing literature, and their implications for future educational practices and research.

### 5.1 The impact of cognitive activation teaching strategies on health behaviors and physical performance

The findings from this study reveal that cognitive activation teaching strategies (CATS) have a significant positive impact on health behaviors and physical performance in physical education (PE). These results underscore the effectiveness of CATS in fostering healthier lifestyles and enhancing physical fitness among students. Physical education differs from traditional academic subjects due to its emphasis on physical activity and health, making the integration of cognitive activation teaching strategies in PE particularly noteworthy and impactful. Previous research has extensively documented the benefits of CATS in subjects such as math and science, and our study extends this evidence to the domain of physical education, demonstrating the versatility and comprehensive impact of these strategies across diverse educational contexts (Li et al., 2021; Montague, 1997; Trudeau and Shephard, 2008).

The positive relationship between CATS and health behaviors suggests that engaging students in cognitively stimulating activities encourages them to adopt healthier lifestyles. This finding aligns with existing literature indicating that when students are intellectually engaged, they are more likely to internalize the benefits of healthy behaviors (Coimbra et al., 2020). Our qualitative data provide further support for these findings. Teachers in our study reported that integrating discussions about the benefits of physical activities and healthy eating into their PE lessons significantly increased students’ interest in adopting these behaviors. One teacher noted, “When we discuss how different exercises and diets affect their health, students become more conscious of their choices and more likely to engage in healthy behaviors.” Another teacher mentioned, “Students started asking more questions about nutrition and exercise routines after we implemented these discussions, showing a clear shift in their awareness and interest in health”.

These observations are supported by other research, such as the study by Trudeau and Shephard (2008), which found that integrating physical activity into the school curriculum positively affects students’ physical fitness and academic performance. Additionally, Donnelly et al. (2016) reported that physical fitness and physical activity interventions can benefit children’s cognitive functioning and academic achievement, emphasizing the dual benefits of CATS in both cognitive and physical domains.

In conclusion, the significant positive impacts of cognitive activation teaching strategies on health behaviors and physical performance in PE highlight their value in this unique educational context. By promoting cognitive engagement, these strategies help students internalize healthy habits and improve their physical fitness.

### 5.2 The mediating role of classroom engagement

This study concludes that classroom engagement significantly mediates the relationship between cognitive activation teaching strategies (CATS) and student outcomes in physical education (PE), consistent with the Stimulus-Organism-Response (SOR) theory. According to SOR theory, positive stimuli such as CATS influence students’ internal states, leading to corresponding positive behaviors. This aligns with previous research demonstrating that engaging teaching strategies improve students’ internal engagement and subsequent behavioral outcomes. For example, González-Peño et al. (2021) found that supportive teaching behaviors positively influenced student engagement in PE, while Errisuriz et al. (2021) showed that high-quality teacher engagement behaviors were associated with improved student physical activity outcomes. Similarly, Ulaywi (2021) highlighted the effectiveness of cognitive activation strategies in fostering better learning environments and student outcomes in other educational contexts.

Further supporting these conclusions, our qualitative data from interviews and observations provide deeper insights into the mechanisms at play. Teachers reported that integrating CATS into PE lessons led to noticeable increases in student engagement and participation. One teacher noted, “Students are more attentive and eager to participate when they are presented with challenging and thought-provoking tasks.” Observations confirmed that students involved in cognitively stimulating activities were more likely to exhibit sustained attention and increased effort in their physical activities. Another teacher highlighted, “When students understand the purpose and benefits of the exercises, they are more committed and perform better.” These qualitative insights reinforce the quantitative findings by illustrating how CATS effectively foster classroom engagement, which in turn enhances both physical performance and health behaviors. Studies like those by Medina et al. (2023) and Aji et al. (2020) also emphasize the positive impact of innovative teaching strategies on student engagement and performance.

Comparing these findings to previous literature, Buchanan et al. (2013) demonstrated the effectiveness of positive behavior support in enhancing student engagement in PE, while Bevans et al. (2010) highlighted the role of instructional determinants in fostering student engagement. These earlier studies support the notion that well-structured and engaging teaching strategies are crucial for improving student outcomes.

In conclusion, the significant positive impacts of cognitive activation teaching strategies on classroom engagement in PE highlight their critical role in enhancing student outcomes. By fostering behavioral engagement, these strategies bridge instructional methods and learning outcomes, promoting both cognitive and physical development.

### 5.3 The mediating role of self-efficacy

Our study demonstrates that self-efficacy significantly mediates the relationship between cognitive activation teaching strategies (CATS) and student outcomes in physical education (PE). Self-efficacy encompasses an individual’s belief in their capacity to execute behaviors necessary for specific performance attainments. Elevated self-efficacy enhances motivation, persistence, and resilience, which are crucial for learning and performance. The results indicate that CATS boost students’ self-efficacy by presenting them with challenging yet attainable tasks, thereby fostering a sense of competence and confidence. This aligns with existing research, which suggests that students with high self-efficacy are more likely to engage in academic activities, exert effort, and persist despite difficulties, ultimately leading to superior academic and physical outcomes (Li et al., 2021; Hsu et al., 2022).

Qualitative data from interviews and observations further illuminate how self-efficacy mediates the effects of CATS. Teachers reported significant changes in students’ confidence and participation over the course of a semester. One teacher described a student who initially struggled with self-doubt and hesitancy in physical activities. “At the beginning of the semester, this student was reluctant to try new exercises and often gave up easily. However, as we implemented more cognitively challenging tasks, I noticed a remarkable transformation. By mid-semester, the student began to approach tasks with more confidence and perseverance. By the end of the term, they were actively participating, even leading group activities and encouraging peers.” Another teacher highlighted similar improvements in a different student: “One of my students was very reserved and rarely participated at the start. Through consistent exposure to CATS, this student gradually gained confidence. They started setting personal goals and tracking their progress, which was something they had never done before. By the end of the semester, their physical performance had improved significantly, and they were much more engaged in class.” These examples vividly illustrate the positive changes in self-efficacy facilitated by CATS, highlighting how these strategies help students overcome initial barriers and develop a stronger sense of capability and engagement in physical activities (García-Hermoso et al., 2020; Lu et al., 2022).

Comparing this with previous findings, Invernizzi et al. (2019) found that a multi-teaching styles approach in PE can improve fitness levels, motor competence, and enjoyment among primary school children. Similarly, González-Peño et al. (2021) observed that teaching behaviors positively influence student engagement in PE, which supports the notion that instructional methods significantly impact student outcomes by enhancing their self-efficacy and engagement.

In summary, the significant positive impacts of cognitive activation teaching strategies on self-efficacy in PE underscore their effectiveness in improving student outcomes. Among the mediators studied, self-efficacy emerged as the most influential, highlighting its pivotal role in linking instructional methods with learning results. By fostering a robust sense of competence and confidence, CATS aid students in adopting healthy habits and enhancing physical fitness (Ekatushabe et al., 2021; Zheng et al., 2020).

### 5.4 Non-significant findings on emotional regulation

The data indicate that emotional regulation neither significantly mediates the relationship between cognitive activation teaching strategies (CATS) and student outcomes in physical education (PE), nor influences health behaviors. This lack of significant impact calls for a detailed discussion. Despite extensive research in other academic domains suggesting that emotional regulation is influenced by engaging and cognitively stimulating tasks, this study found no significant impact of CATS on emotional regulation in PE. Studies in subjects like mathematics and science have shown that emotionally supportive environments and challenging cognitive tasks can enhance students’ emotional regulation (Cruz et al., 2020). However, our findings do not corroborate these results in the context of PE.

Qualitative data support this discrepancy. Teachers reported that while CATS were effective in enhancing cognitive and physical engagement, they did not observe notable changes in students’ emotional regulation. One teacher commented, “Students are more focused and participate actively due to the challenging tasks, but their emotional responses to success or failure remain largely unchanged.” This suggests that while CATS can stimulate cognitive and physical aspects, they may not sufficiently address the emotional dimension within the context of PE (Simonton et al., 2017).

Furthermore, the study found no significant relationship between emotional regulation and health behaviors. In the Chinese educational context, the development of health behaviors appears to be more influenced by external factors such as parental guidance, school policies, and societal norms rather than by students’ emotional regulation. This contrasts with findings from Western contexts where emotional regulation plays a more significant role in shaping health behaviors (Hsu et al., 2022). Teacher insights provide additional context. One teacher noted, “Students’ decisions regarding their health habits, such as diet and exercise outside school, seem more influenced by their parents and social environment than by their ability to manage emotions.” This highlights that the motivational and behavioral drivers for health behaviors among Chinese students may not be directly linked to emotional regulation, but rather to cultural and contextual factors that prioritize compliance and external reinforcement over internal emotional management.

Comparative analysis with studies from different contexts supports the complexity of emotional regulation in educational settings. For instance, a study on the emotional and cognitive regulation of students during the COVID-19 lockdown found that the lockdown situation heightened the importance of cognitive strategies in managing emotional well-being, emphasizing the role of external situational factors (Simonton and Garn, 2020; Bakul and Heanoy, 2021). Similarly, research on pre-service teachers showed that simulation-based learning significantly helped in the development of emotional self-regulation, but such methods are rarely used in PE (Jong et al., 2012). This indicates that the lack of significant impact of CATS on emotional regulation in PE may be due to differences in the nature of tasks and the emotional engagement they elicit.

In summary, the non-significant findings on emotional regulation underscore the complexity of its role within the context of PE and health behaviors. While CATS enhance cognitive and physical engagement, they do not significantly influence emotional regulation. Additionally, emotional regulation does not appear to mediate the development of health behaviors among Chinese students, suggesting that cultural and contextual factors play a more significant role in shaping these behaviors. This emphasizes the need for culturally tailored approaches to promoting health behaviors.

### 5.5 The mediating role of motivation

This study reveals that motivation significantly mediates the relationship between cognitive activation teaching strategies (CATS) and student outcomes in physical education (PE), aligning with numerous studies grounded in Self-Determination Theory (SDT). Research consistently shows that fulfilling students’ needs for autonomy, competence, and relatedness through engaging instructional strategies enhances intrinsic motivation (Leo et al., 2020; Estevan et al., 2020). For example, research indicates that a need-supportive teaching style in PE promotes students’ self-determined motivation and intention to engage in physical activities outside the school context (Kelso et al., 2020).

Despite the positive impact of motivation on physical performance, it did not translate into improved health behaviors. This finding can be attributed to the nature of students’ motivation in PE, which appears to be more competence-oriented rather than lifestyle-oriented. Previous research has indicated that while students may develop motivation to improve their physical skills and performance within the structured environment of PE classes, this motivation does not necessarily extend to adopting healthier behaviors outside the classroom (Simonton et al., 2017; Behzadnia, 2020).

Teacher feedback supported this observation. One teacher noted, “Students are keen to improve their athletic abilities and often set goals related to physical performance, but this enthusiasm does not seem to carry over to their daily health habits.” This suggests that the motivation fostered by CATS is primarily directed toward enhancing physical competence rather than promoting a broader awareness of health behaviors. The lack of impact on health behaviors may also be due to the need for a more comprehensive support system and continuous reinforcement beyond the classroom, which CATS alone cannot provide.

In summary, while motivation plays a critical role in enhancing physical performance through cognitive activation teaching strategies, its influence on health behaviors is limited. This discrepancy highlights the context-specific nature of motivation in PE, where students’ focus remains on improving their physical abilities rather than adopting healthier lifestyles. To effectively promote health behaviors, additional strategies that extend beyond the PE classroom and address the broader aspects of students’ lives are necessary.

## 6 Conclusion

This study demonstrates the significant impact of cognitive activation teaching strategies (CATS) on enhancing self-efficacy, motivation, and classroom engagement in physical education (PE). By fulfilling students’ needs for autonomy, competence, and relatedness, CATS increase intrinsic motivation and active participation in PE activities. Teacher interviews and classroom observations confirm these findings, showing that strategies such as goal-setting, interactive tasks, and constructive feedback foster a dynamic learning environment.

However, CATS had limited influence on emotional regulation and health behaviors, indicating that these areas may need additional support beyond the classroom. This highlights the necessity for comprehensive approaches that address both educational and lifestyle factors. Integrating CATS into PE curricula significantly benefits student motivation, engagement, and physical performance, contributing to their overall development. Future research should further explore CATS’ effects in various educational contexts and find additional ways to support emotional regulation and health behaviors in students.

A key limitation is that, for privacy and policy-sensitivity reasons, we did not collect parent-reported or device-based data on pupils’ after-school physical activity; future work should incorporate such measures to disentangle classroom effects from lifestyle influences on emotional regulation and health behaviors.

## Data Availability

The raw data supporting the conclusions of this article will be made available by the authors, without undue reservation.
